# Clinical, Demographic, and Virological Predictors of Hospital Admission in Patients with Acute Viral Respiratory Infections: A Retrospective Observational Study

**DOI:** 10.3390/v18010135

**Published:** 2026-01-21

**Authors:** Karolina Akinosoglou, Nikolaos Theofanis, Konstantinos Asimos, Michail Michailidis, Despoina Papageorgiou, Eleni Polyzou, Charalambos Gogos

**Affiliations:** 1 Department of Medicine, University of Patras, Rio, 26504 Patra, Greece; dspn.pap96@gmail.com (D.P.); polyzou.el@gmail.com (E.P.); cgogos@med.upatras.gr (C.G.); 2 Department of Internal Medicine, University General Hospital of Patras, Rio, 26504 Patras, Greece; 3 Division of Infectious Diseases, Department of Internal Medicine, University General Hospital of Patras, Rio, 26504 Patras, Greece; 4 School of Science and Technology, Hellenic Open University, 26335 Patras, Greece; std535334@ac.eap.gr; 5 Metropolitan General Hospital of Athens, 15562 Athens, Greece; asimos.kon@gmail.com (K.A.); michailidismichail8@gmail.com (M.M.)

**Keywords:** respiratory tract infections, hospital admission, Respiratory Syncytial Virus, Severe Acute Respiratory Syndrome Coronavirus 2, influenza A, influenza B, human rhinovirus, adenovirus, enterovirus, human metapneumonovirus, coronavirus, parainfluenza virus

## Abstract

Background: Viral respiratory tract infections (RTIs) frequently lead to emergency department (ED) presentations and hospital admissions, particularly among older adults and individuals with underlying health conditions. Identifying patients at increased risk for hospitalization is essential for optimizing triage and resource allocation. This study aimed to determine independent demographic, clinical, and virological predictors of hospital admission among adults presenting with confirmed viral RTIs. Methods: A retrospective cohort study was conducted at a tertiary hospital between September 2022 and May 2024. Adult patients with molecularly confirmed viral RTIs were included. Demographic, clinical, and microbiological data were extracted from electronic medical records. Predictors of admission were assessed using univariate and multivariate logistic regression. Results: Among 311 patients, 147 (47.3%) required hospitalization. Hospitalized patients were significantly older and more likely to present with fever, cough, tachypnea, dyspnea, chest pain, comorbidities, and lower or mixed respiratory tract infections (all *p* < 0.001). In multivariate analysis, older age, fever, cough, and lower or mixed RTIs were strong independent predictors of admission. Several viral pathogens, including human rhinovirus, non–SARS-CoV-2 coronaviruses, influenza A, and parainfluenza virus, were associated with reduced odds of hospitalization. Conclusions: Age, comorbidity burden, and lower respiratory tract involvement are key determinants of hospitalization in viral RTIs. Integrating clinical and virological data may improve risk stratification and guide ED triage during seasonal and emerging respiratory virus activity.

## 1. Introduction

Respiratory tract infections (RTIs) caused by viral pathogens still pose a persistent challenge to public health, driven mainly by their rapid transmission, seasonal outbreaks and considerable strain on healthcare infrastructure. Global epidemiological analyses consistently demonstrate that RTIs remain highly prevalent worldwide, with viral pathogens accounting for a substantial proportion of incident cases [1]. Large-scale global estimates indicate that more than 340 million new cases are reported globally in a single year, with a greater share occurring in low-income and lower-middle-income settings [1]. They still remain one of the major public health problems, given that only during the 2022–2023 season, over 1.5 million laboratory-confirmed influenza cases and more than 250,000 cases of Respiratory Syncytial Virus (RSV) were reported across the European Union/European Economic Area countries, according to surveillance data of the European Centre for Disease Prevention and Control (ECDC) [2].

Although most cases are self-limiting, a considerable subgroup of patients develops complications like bronchiolitis, viral pneumonia or exacerbations of asthma or Chronic Obstructive Pulmonary Disease (COPD), requiring inpatient care [3]. These infections are among the leading causes not only of hospitalization, but also of morbidity, most pronounced in older patient groups and in groups of patients with coexisting chronic diseases [3]. In more severe forms, viral respiratory infections may progress to acute respiratory failure or acute respiratory distress syndrome (ARDS), particularly among elderly individuals, immunocompromised patients, and those with chronic cardiopulmonary conditions; furthermore, concurrent bacterial co-infection can exacerbate respiratory compromise and contribute to poorer clinical outcomes [4].

In greater detail, viral pathogens including RSV, influenza (type A and B), and Severe Acute Respiratory Syndrome Coronavirus 2 (SARS-CoV-2) contribute significantly to global morbidity and mortality particularly among vulnerable populations such as infants, older adults or individuals with chronic comorbidities or immunosuppression [3]. The viral strains of Influenza A and B cause about 700.000 deaths annually all over the world [5] and the pandemic of SARS-CoV-2 has led to the death over 7 million people globally since 2020 [6]. Their exceptionally fast contagion via droplet, contact and airborne transmission routes facilitates widespread outbreaks, overwhelming primary care and hospital services during peak seasons [7]. However, it is important to note that the abrupt collapse in circulation of influenza A, influenza B, and RSV during the COVID-19 restriction period, the loss of their usual seasonal peaks, the sudden off-season resurgence, particularly of RSV, in 2021, and the overall instability in viral transmission patterns all translated into substantial challenges for healthcare systems [8]. Even when global studies identify upper RTIs (URTIs) as having minimal associated mortality, these infections represent a substantial burden on quality of life driven by high incidence and disability [9]. As a result, they exert a notable economic toll, reflected in higher public healthcare expenditures, absenteeism in the labour force, and overall productivity decline. For a non-hospitalized case of RSV infection, the mean direct cost is estimated at €26.4, while the mean indirect cost, primarily due to lost workdays, is €4.4 across European Union countries [10].

To mitigate the burden of these infections, comprehensive surveillance, targeted immunization efforts and early detection of those patients at elevated risk for hospitalization are required. Based on the above, it becomes evident that correlating demographic and clinical aspects is essential for the timely identification of patients who require hospital care. The present study aims to delineate independent risk factors for hospitalization in adults with confirmed viral respiratory infections, utilizing multivariable logistic regression to evaluate the impact of age, sex, viral agent, presenting symptoms and comorbidities.

## 2. Materials and Methods

### 2.1. Study Design and Setting

This was a single-centre retrospective cohort study, utilizing the electronic medical record system of Metropolitan General Hospital, a tertiary private healthcare facility located in Athens, Greece, from 1 September 2022 to 1 May 2024 (study period). The primary outcome of this study was hospital admission status, defined as whether the patient was admitted for impatient care following the clinical and laboratory evaluation in the Emergency Department (ED) or not. This binary outcome (admitted vs. not admitted) served as proxy for clinical severity and guided the identification of risk factors associated with the need for hospitalization. Hospital admission decisions were based on clinical severity and followed the recommendations of the Hellenic Society for Infectious Diseases. Criteria for admission included hypoxemia (peripheral oxygen saturation (SpO_2_) < 92% on room air), respiratory distress, hemodynamic instability, altered mental status, and the presence of significant comorbidities. Severity assessment tools such as the Confusion, Urea, Respiratory rate, Blood pressure, age ≥ 65 years (CURB-65) score [11] and the Pneumonia Severity Index/Patient Outcomes Research Team (PSI/PORT) score [12] were used in the ED to support clinical decision-making. Patients meeting criteria for severe disease, according to American Thoracic Society/Infectious Diseases Society of America (ATS/IDSA) guidelines [13], were admitted to the intensive care unit (ICU) when indicated. Major criteria included the need for invasive or non-invasive mechanical ventilatory support due to acute respiratory failure and/or the requirement for vasopressor therapy to maintain adequate arterial blood pressure. Minor criteria included respiratory rate ≥ 30 breaths per minute, PaO_2_/FiO_2_ ratio ≤ 250, multilobar pulmonary infiltrates, altered mental status or confusion, uremia (blood urea nitrogen > 40 mg/dL), leukopenia (<4000 cells/μL) attributable to infection, thrombocytopenia (platelet count < 100,000/μL), hypothermia (core temperature < 36.8 °C), hypotension requiring aggressive fluid resuscitation, and need for resuscitation.

The study aimed to determine which variables, including age, sex, presenting symptoms (fever, dyspnea, tachypnea, chest pain), comorbidities, and the specific viral pathogen detected via FilmArray^®^ multiplex PCR, were independently associated with hospital admission. No secondary outcomes were predefined, as the study focused exclusively on admission-related predictors in the context of confirmed viral respiratory infections.

### 2.2. Study Population and Inclusion Criteria

The study population consisted of adult patients (≥18 years old) who presented to the ED of the Metropolitan General Hospital and were diagnosed with a confirmed viral respiratory infection using the FilmArray^®^ multiplex PCR panel, as per standard protocol [14]. Only patients with a positive result for at least one respiratory pathogen were included in this study. Patients with evidence of bacterial infection—defined as a positive bacterial culture and/or detection of bacterial pathogens on the FilmArray^®^ panel—were excluded. Additional exclusion criteria included incomplete medical records, age under 18 years, absence of confirmatory FilmArray^®^ testing, and repeat visits for the same illness episode to prevent duplication of cases.

The anatomical site of infection was classified based on clinical presentation and radiologic findings. URTI was defined as the presence of respiratory symptoms confined to the upper airway, in the absence of radiographic evidence of pneumonia. LRTI was defined by clinical features suggestive of lower respiratory tract involvement, in combination with radiologic findings consistent with pneumonia on chest imaging. Mixed infection was defined as the coexistence of upper respiratory tract symptoms and radiologic evidence of lower respiratory tract involvement.

After application of all inclusion and exclusion criteria, a total of 311 patients were included in the final analysis (Supplementary Figure S1).

### 2.3. Data Collection

Patient data were retrospectively retrieved from the electronic medical records (EMR) of Metropolitan General Hospital. Data extraction was initially performed by the hospital’s IT department using a standardized extraction protocol, under the supervision of the research team to ensure consistency across records and minimize selection bias. Subsequently, data accuracy and completeness were independently reviewed by three investigators. Any discrepancies identified during data review were resolved by consensus through discussion among the investigators, with reference to the original medical records. This process was implemented to ensure data quality and consistency across records.

### 2.4. Sampling and Microbiological Diagnosis

Microbiological confirmation of respiratory pathogens was obtained through the FilmArray^®^ multiplex Polymerase Chain Reaction (PCR) Respiratory Panel of nasopharyngeal swabs obtained upon presentation. The FilmArray^®^ Respiratory panel (RP) targets 20 pathogens including 17 viruses and subtypes and three bacteria. This method has a fully automated sample-to-answer workflow and the results of the test are available in approximately 1 h [14].

### 2.5. Comorbidity Evaluation

Assessment of participants’ comorbidities was conducted systematically, allowing patients to be classified into three distinct categories according to the number of chronic medical conditions they presented. This classification was based both on the medical history obtained during triage in the ED and on the review of medical records for those who had previously visited or been hospitalized at the Metropolitan Hospital. Specifically, the assessment considered the presence of chronic heart disease, chronic kidney disease, diabetes mellitus, COPD, immunosuppression (due to coexisting neoplastic disease or other causes), as well as other chronic pathological conditions not included in the aforementioned categories. Patients were categorized according to comorbidity burden based on the number of documented chronic conditions (none, one, or two or more). This approach is consistent with the widely used definition of multimorbidity as the presence of two or more chronic conditions [15] and with prior large-scale epidemiological studies that analyze comorbidity burden according to the number of conditions [16,17]. More specifically, patients were grouped as follows: the “No Comorbidities” category, which included individuals who neither reported any chronic illness nor had any such condition documented in their electronic medical record); the “Comorbidity Category 1,” comprising patients with one of the listed chronic conditions; and the “Comorbidity Category 2,” which included patients with two or more of the aforementioned conditions.

### 2.6. Statistical Analysis

All statistical analyses were performed using SPSS v. 29.0 (IBM Corp, Armonk, NY, USA). Descriptive statistics were used to summarize the demographic and clinical characteristics of the study population. Categorical variables were expressed as frequencies and percentages, while continuous variables were reported as means with standard deviations or medians with interquartile ranges, depending on distribution. Comparisons between admitted and non-admitted patients were conducted using Chi-square test or Fisher’s exact test for categorical variables and independent samples *t*-test or Mann–Whitney U test for continuous variables. When comparisons involved more than two groups, a one-way ANOVA was applied for normally distributed variables. If the ANOVA demonstrated a statistically significant overall effect, post hoc analyses using the Bonferroni correction were performed to determine pairwise differences. For non-normally distributed variables with more than two groups, the Kruskal–Wallis test was applied, followed by Dunn’s post hoc test where appropriate. To identify independent predictors of hospital admission, univariate logistic regression was initially applied to each variable. Variables with a *p*-value < 0.10 were subsequently entered into a multivariate logistic regression model using a stepwise forward selection method. Odds ratios (ORs) with 95% confidence intervals (CIs) were calculated, and statistical significance was set at *p* < 0.05. Model performance and goodness-of-fit were assessed using the Hosmer–Lemeshow test and Nagelkerke R^2^.

### 2.7. Study Ethics

The study was conducted in accordance with the principles outlined in the Declaration of Helsinki and adhered to national ethical standards for retrospective observational research in line with Good Clinical Research Practice. Approval was obtained from the Ethics Committee of Metropolitan General Hospital (788/15.04.2024) prior to data collection. All patient data was fully anonymized before analysis. No identifiable information was accessed or stored. Given the retrospective nature of the study and the use of routinely collected clinical data, informed consent was waived by the Ethics Committee.

## 3. Results

### 3.1. Patients Characteristics

In total, 311 patients were included in the analysis, of whom 147 (47.3%) were hospitalized and 164 (52.7%) were discharged. The study flowchart is presented in Supplementary File.

A number of demographic and clinical characteristics differed significantly between the two groups. Hospitalized patients were significantly older than non-hospitalized patients (median age 69 vs. 44 years, *p* < 0.001), whereas males were more frequently hospitalized (*p* = 0.03) (Table 1).

Hospitalized individuals exhibited markedly more severe presentations. Fever, cough, tachypnea, dyspnea, and chest pain were all significantly more common among admitted patients (all *p* < 0.001), while these symptoms were rare among discharged individuals. Comorbidity burden also differed: hospitalized patients had a higher prevalence of both single and multiple comorbidities, while discharged patients were mostly without underlying conditions (*p* < 0.001). Additionally, lower or mixed RTIs occurred more often among hospitalized patients, whereas URTIs predominated among those discharged (*p* < 0.001).

### 3.2. Viral Etiology

**Figure 1:**
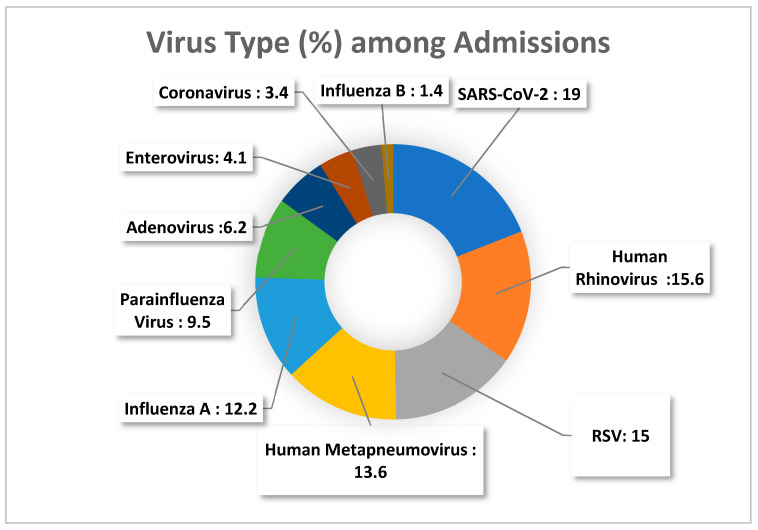
Distribution of respiratory viruses among hospitalized patients.

**Figure 2:**
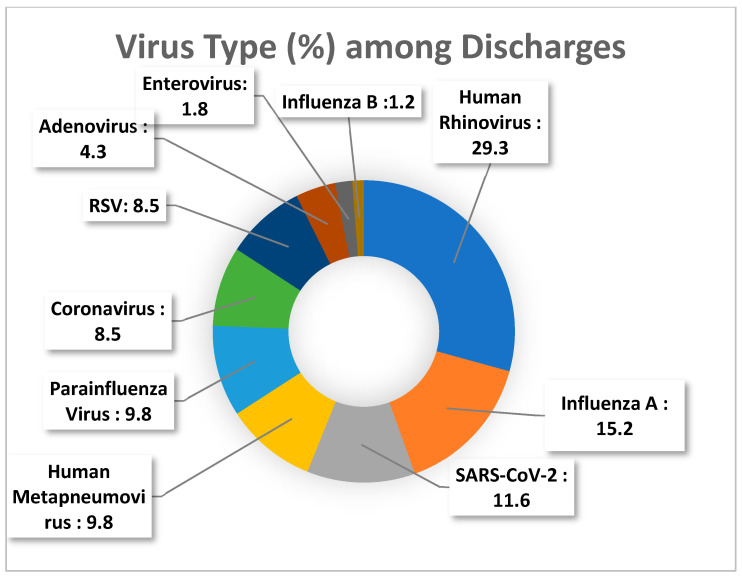
Distribution of respiratory viruses among discharged patients.

The distribution of viral pathogens among admitted and discharged patients is presented in Figure 1 and Figure 2, respectively.

Among the detected viral pathogens, most did not show statistically significant differences between hospitalized and discharged patients. The proportions of adenovirus, human metapneumovirus (hMPV), influenza A, influenza B, parainfluenza virus, enterovirus, and RSV were comparable across the two groups (*p* > 0.05 for all). Detection of coronaviruses and SARS-CoV-2 also did not differ significantly between admission statuses, although both trends approached significance (*p* = 0.059 and *p* = 0.067, respectively).

The only virus showing a statistically meaningful difference was human rhinovirus (HRV), which was more frequently detected among discharged patients compared with hospitalized patients (29.3% vs. 15.6%, *p* = 0.004), suggesting an association with a milder clinical course.

### 3.3. Clinical Characteristics According to Detected Virus

When comparing clinical characteristics across the different viral infection groups, several statistically significant differences were observed (Table 2). Fever showed notable variation between viruses (*p* = 0.021), with higher frequencies seen among patients infected with influenza B, adenovirus, parainfluenza virus, and enterovirus compared with other viral groups. Tachypnea also differed significantly across viruses (*p* = 0.016), occurring most often in patients with enterovirus, RSV, or parainfluenza infections.

In contrast, cough, dyspnea, and chest pain did not differ significantly across viral groups (*p* > 0.05 for all). Comorbidity profiles similarly showed no statistically significant variation, whether considering absence of comorbidities, single comorbidity, or multiple comorbidities. The distribution of URTIs, LRTIs, and mixed RTIs was also comparable among all viral categories (*p* > 0.05 for all comparisons). The sex distribution likewise showed no meaningful differences across viral groups (*p* = 0.781).

### 3.4. Analysis of Predictors of Hospital Admission

Table 3 presents the univariate and multivariate logistic regression analyses for factors associated with hospital admission.

Among population characteristics, male gender showed higher odds of hospitalization in univariate analysis (OR 1.660, 95% CI 1.048–2.630, *p* = 0.031), but this association was not significant in the multivariate model (adjusted OR 1.101, 95% CI 0.577–2.103, *p* = 0.770). Age over 60 years was significantly associated with admission in both analyses, with an OR of 5.961 (95% CI 3.542–10.033, *p* < 0.001) in univariate analysis and an adjusted OR of 6.110 (95% CI 3.046–12.256, *p* < 0.001) in the multivariate model.

Regarding clinical manifestations, fever was associated with higher odds of hospitalization (OR 19.841, 95% CI 8.708–45.207, *p* < 0.001), and this remained significant in the multivariate model (adjusted OR 20.502, 95% CI 7.067–59.475, *p* < 0.001). Cough showed a similar pattern, with an unadjusted OR of 12.277 (95% CI 5.358–28.132, *p* < 0.001) and an adjusted OR of 3.999 (95% CI 1.280–12.494, *p* = 0.017), respectively.

For site of infection, lower respiratory tract infection and mixed URTIs and LRTIs were compared with URTIs as the reference category. In univariate analysis, lower infection had an OR of 3.785 (95% CI 1.549–9.250, *p* = 0.004) and mixed infection had an OR of 6.624 (95% CI 1.424–30.807, *p* = 0.016). Both remained significant in the multivariate model, with adjusted ORs of 11.109 (95% CI 3.609–34.196, *p* < 0.001) and 22.285 (95% CI 4.318–115.002, *p* < 0.001), respectively.

Regarding viral pathogens, adenovirus, hMPV, influenza A, influenza B, parainfluenza virus, enterovirus, and RSV did not show significant associations with hospitalization in univariate analysis. HRV showed lower odds of admission (OR 0.448, 95% CI 0.257–0.783, *p* = 0.005), while SARS-CoV-2 showed a borderline association (OR 1.796, 95% CI 0.955–3.375, *p* = 0.069).

In the multivariate model, coronavirus (adjusted OR 0.066, 95% CI 0.011–0.387, *p* = 0.003), HRV (adjusted OR 0.235, 95% CI 0.086–0.644, *p* = 0.005), influenza A (adjusted OR 0.261, 95% CI 0.084–0.808, *p* = 0.020), and parainfluenza virus (adjusted OR 0.151, 95% CI 0.041–0.560, *p* = 0.005) were significantly associated with lower odds of hospitalization. Adenovirus (*p* = 0.468), hMPV (*p* = 0.230), influenza B (*p* = 0.559), enterovirus (*p* = 0.824), and RSV (*p* = 0.432) were not significant in the adjusted model. SARS-CoV-2 could not be included in the multivariate analysis due to separation issues. The multivariate logistic regression model demonstrated good calibration, as indicated by a non-significant Hosmer–Lemeshow goodness-of-fit test (χ^2^ = 6.576, df = 8, *p* = 0.583), and showed strong explanatory power with a Nagelkerke R^2^ of 0.586.

## 4. Discussion

This study aimed to identify independent clinical, demographic, and virological predictors of hospital admission among patients presenting with acute viral respiratory infections in the ED. Through a structured analysis incorporating both univariate and multivariate logistic regression, several independent factors were identified as significantly associated with hospitalization. These findings contribute to the growing body of evidence on risk stratification in respiratory infections and offer practical guidance for emergency department triage and public health preparedness.

Patients admitted to the hospital tended to be older (median: 69 years, IQR: 28 years) compared with the discharged patients (median: 44 years IQR = 23 years). Age emerged as a powerful independent predictor of hospital admission with an adjusted OR of 6.110 (3.046–12.256, *p* < 0.001), a trend that has also been demonstrated in other studies [18,19,20]. This finding is in line with extensive epidemiological data demonstrating that older adults are disparately affected by severe respiratory infections, largely due to immunosenescence, chronic comorbidities, reduced mucociliary clearance, and diminished physiological reserve [21,22,23]. From a pathophysiological perspective, ageing leads to dysregulated immune responses, including impaired antiviral defences, reduced mucosal immunity, and exaggerated inflammatory reactions, which collectively heighten susceptibility to severe respiratory disease [24,25,26]. Furthermore, consistent with previous reports, RSV and COVID-19 were predominantly detected in older individuals in our cohort, whereas adenovirus and influenza B were more common among younger adults, suggesting age-dependent patterns in viral susceptibility and pathogenicity [27].

In our study, male gender showed higher odds of hospitalization in univariate analysis (OR 1.660, 95% CI 1.048–2.630, *p* = 0.031). However, this association was not sustained in the multivariate model (adjusted OR 1.101, 95% CI 0.577–2.103, *p* = 0.770), indicating that the apparent effect of gender was likely confounded by other variables. Although gender differences in respiratory infection outcomes are variably reported, some studies suggest that males may have higher risk of severe disease and hospitalization due to differences in immune response and comorbidity burden [28,29].

In the context of coexisting chronic health conditions, comorbidity was strongly associated with hospital admission in our cohort. Compared with non-hospitalized patients, hospitalized individuals were far more likely to have at least one chronic condition particularly cardiovascular disease, diabetes mellitus, chronic kidney disease, and chronic lung disease. Among those admitted, 72.1% had no comorbidities, 17.7% had one comorbidity, and 10.2% had two or more. In contrast, nearly all non-hospitalized patients (99.4%) had no comorbidities, with only 0.6% reporting a single comorbidity and none reporting two or more. This overall distribution differed significantly between the two groups (*p* < 0.001). These findings come as no surprise, as multiple studies consistently demonstrate that presence of co-morbidities impair immune responses, increase susceptibility to complications, and are related to higher rates of hospitalization and poor outcomes [21,30].

As for the patient clinical presentation in the ED, symptoms such as dyspnea, tachypnea, and chest pain were exclusively observed among admitted patients and for this reason were excluded from the multivariate model due to lack of variability. Nonetheless, these symptoms are well-established markers of severe respiratory impairment and consequently, correlated to high risk for hospitalization [21]. This is further supported by evidence from primary care surveillance research, which identifies shortness of breath and other signs of respiratory distress as fundamental severity markers for acute respiratory infections [31]. Their presence remains clinically meaningful and aligns with established triage criteria such as the CURB-65 and PSI [32]. In our study, fever was significantly associated with an increased likelihood of hospital admission. In univariate analysis, fever demonstrated a high odds ratio (OR = 19.84, 95% CI: 8.71–45.21, *p* < 0.001). This association remained after adjustment for demographic and clinical factors, with an adjusted OR of 20.50 (95% CI: 7.07–59.48, *p* < 0.001). However, the wide CIs suggest instability of the model, and therefore these estimates should be interpreted with caution. The absence of fever in adults with viral respiratory infections has been linked to delayed diagnosis, reduced testing in the ED, less antiviral treatment, longer length of stay, and worse clinical outcomes, including higher inpatient mortality in cohorts [20,33,34]. While mild fever may confer antiviral benefits through suppression of viral replication and enhancement of host immune responses, fever becomes less advantageous at higher temperatures due to increased metabolic demand and potential tissue damage [35]. Thus, both the presence and absence of fever carry important diagnostic and prognostic implications in adult viral infections.

Interestingly, specific respiratory pathogens may predispose to more severe symptomatology [36]. In particular, hMPV was associated with elevated rates of dyspnea and shortness of breath in our study, consistent with previous reports [7]. Despite the notable clinical complications associated with hMPV, the virus itself was not recognized as an independent predictor of hospitalization.

The clinical diagnosis of LRTI, either isolated or in combination with upper respiratory tract symptoms, was strongly associated with hospital admission. Using URTI as the reference category, LRTI demonstrated a markedly increased likelihood of admission in the multivariate model (adjusted OR 11.109, 95% CI 3.609–34.196, *p* < 0.001). Similarly, mixed URTI and LRTI was associated with an even higher risk of hospitalization (adjusted OR 22.285, 95% CI 4.318–115.002, *p* < 0.001). This is expected, given that a total number of 90 million hospitalizations due to viral LRTI has been recorded globally, during the period from 2010 to 2019, and the average overall global incidence of viral LRTI hospitalization was 170 per 10.000 person-years during the period from 2010 to 2021. Meanwhile, the number of LRTI cases was 880 million in 2010–2019 and increased dramatically to 4 billion cases in 2020–2021 [37]. These findings are consistent with reports demonstrating that LRTIs are associated with significant morbidity and often necessitate hospitalization, regardless of underlying etiology [38]. Although substantial global progress has been made in reducing the incidence and mortality of LRTIs, the absolute number of cases and deaths remains high, underscoring the continued public health importance of LRTIs and the need for sustained prevention and control efforts [39].

The role of viral etiology in predicting admission varied across pathogens. While some viruses such as RSV, Influenza A, and SARS-CoV-2 were more prevalent among admitted patients, only a few retained independent associations in the adjusted model. According to the Centre for Disease Control (CDC), in the United States, SARS-CoV-2, Influenza and RSV were the most common viral factors in cases of Viral Respiratory Tract hospitalizations [40]. Moreover, the frequency of these hospital admissions exhibited an upward trend from mid-December 2024 to mid-March 2025, indicating a seasonal distribution of hospitalization rates [40]. With respect to the viral agents that were independent predictors in our model, HRV (adjusted OR = 0.235, 95% CI: 0.086–0.644, *p* = 0.005), non-SARS-CoV-2 coronaviruses (adjusted OR = 0.066, 95% CI: 0.011–0.387, *p* = 0.003), and Parainfluenza virus (adjusted OR = 0.151, 95% CI: 0.041–0.560, *p* = 0.005) were each associated with significantly lower odds of hospital admission. In the past, HRV was predominantly associated with self-limiting URTIs. However, it is important not to underestimate its clinical significance, as an increasing body of literature highlights its role as a causative agent of severe LRTIs in adults, including exacerbations of COPD and pneumonia [41]. Notably, these severe manifestations can also occur in immunocompetent individuals [42]. The relevance of this observation is further underscored by the high prevalence of HRV-related respiratory infections and the virus’s capacity for rapid and widespread transmission among individuals [43,44]. To our surprise, RSV was not identified as a predictor of hospitalization in our analysis, even though previous studies have demonstrated that RSV infections in older adults are frequently more severe than influenza and associated with higher rates of ICU admission and mortality [27,45,46]. A possible explanation is that variables directly reflecting severe clinical presentation were excluded from the regression model, which may have attenuated the apparent impact of RSV on admission risk. The protective association observed may reflect both biological factors, such as viral tropism and replication kinetics [47,48], and healthcare system dynamics, including clinician perception of severity and admission thresholds [49]. It is also possible that patients infected with these viruses were younger and had fewer comorbidities, although our model adjusted for these variables. These findings support the notion that not all respiratory viruses carry equal prognostic weight and that viral identification may aid in risk stratification and resource allocation.

From a clinical perspective, the integration of demographic, clinical, and virological data can enhance early risk assessment and guide decisions regarding hospital admission, isolation, and monitoring. In settings with limited resources or during seasonal outbreaks, prioritizing patients based on age, comorbidity, and lower respiratory tract involvement may improve outcomes and reduce unnecessary admissions. The identification of viral aetiologies such as Parainfluenza and HRV associated with milder disease may also support outpatient management and reduce diagnostic uncertainty.

From a public health standpoint, these findings may inform the development of predictive algorithms and triage tools that incorporate both host and pathogen factors. Such tools could be integrated into electronic health records or mobile applications to support frontline clinicians in real time. Moreover, understanding the differential impact of viral pathogens on hospitalization risk may guide vaccination strategies, surveillance priorities, and preparedness planning during respiratory virus seasons and pandemics.

This study has several limitations that must be acknowledged. First, being a retrospective single-centre study, the findings may be influenced by the specific clinical practices, admission thresholds, and diagnostic protocols of this private healthcare infrastructure. These significant factors may limit the generalizability of the results to other healthcare settings with different patient populations, resource availability, triage strategies or health insurance coverage. Second, the retrospective design inherently restricts control over data collection and completeness. Key variables such as vaccination or prior immunity status, socioeconomic background, timing of symptom onset, and prior healthcare visits or/and treatments already received were not consistently documented and therefore could not be included in the analysis. Third, symptoms with perfect prediction (e.g., dyspnea, tachypnea, chest pain) were excluded from the multivariate model due to lack of variability, which may underestimate their clinical relevance in real-world triage. In addition, several predictors in the logistic regression analysis, particularly clinical manifestations and infection site categories, were strongly associated with hospital admission, as these features are inherently more likely to be present in patients requiring hospitalization. In addition, some variables, such as LRTIs, mixed upper and LRTIs, and certain viral pathogens, were characterized by small numbers of events, often concentrated in one outcome group. This combination of strong associations, imbalanced distributions, and sparse data reduces the precision of odds ratio estimates and results in wide confidence intervals. These wide intervals reflect uncertainty in the magnitude of the estimated effects rather than invalid or unreliable results. While the direction and statistical significance of these associations remain robust, the exact effect sizes should therefore be interpreted with caution. Fourth, the absence of biomarker data (e.g., CRP, procalcitonin), radiographic findings, and viral load quantification limited the ability to explore pathophysiological mechanisms and refine predictive accuracy. Fifth, the sample size, while adequate for multivariate modelling, may constrain the detection of harder to detect associations, particularly for less prevalent viral pathogens such as influenza B and enterovirus. This reflects the known epidemiological distribution of certain respiratory viruses, as influenza B typically accounts for a smaller proportion of cases compared with influenza A in most seasons, a pattern consistently reported in previous studies and national surveillance data [50,51]. Nevertheless, the limited number of cases for these viruses reduces statistical power and the precision of between-group comparisons. Consequently, analyses according to viral etiology were primarily exploratory, and the corresponding statistical results should be interpreted with caution. Additionally, although comorbidities were quantified, their impact remains heterogeneous due to the diversity of underlying conditions. This study categorized comorbidities solely based on their number, without accounting for the severity of individual conditions, which may have led to an underestimation of the true impact of comorbidities on hospitalization risk. No co-medication data, including the use of immunosuppressive medication, were recorded, limiting the ability to account for important treatment-related modifiers. Lastly, the study did not include longitudinal follow-up, preventing assessment of downstream outcomes such as ICU admission, duration of stay, or mortality. Future prospective, multicenter studies with standardized data collection and broader variable inclusion are recommended to validate and expand upon these findings.

## 5. Conclusions

This study identified key demographic, clinical, and virological factors independently associated with hospital admission among patients presenting with acute viral respiratory infections. Older age, presence of comorbidities, and lower respiratory tract involvement emerged as the most robust predictors of hospitalization, underscoring the importance of host vulnerability and disease severity in triage decisions. In contrast, certain viral aetiologies, such as HRV, non-SARS-CoV-2 coronaviruses, and Parainfluenza virus, were associated with reduced odds of admission, suggesting a milder clinical course and potential for safe outpatient management.

These findings highlight the value of integrating clinical presentation with virological data to enhance risk stratification and guide resource allocation in emergency care settings. The results may inform the development of predictive models and decision-support tools aimed at improving triage accuracy and optimizing patient flow, particularly during seasonal viral outbreaks or pandemics. Future prospective, multicenter studies are needed to validate these associations and explore their applicability across diverse healthcare environments.

## Figures and Tables

**Table 1:** Population characteristics of Hospitalized and Non-hospitalized Patients.

	All Patients (*n* = 311)	Admission (*n* = 147)	Discharged (*n* = 164)	*p*-Value
Demographic characteristics				
Female *n* (%)	191 (61.4)	81 (55.1)	110 (67.1)	***p* = 0.03**
Male *n* (%)	120 (38.6)	66 (44.9)	54 (32.9)	
Age [in years, median (IQR)]	52 (39–72)	69 (50–78)	44 (32–55)	***p* < 0.001**
Clinical manifestations				
Fever *n* (%)	76 (24.4)	69 (46.9)	7 (4.3)	***p* < 0.001**
Cough *n* (%)	59 (19)	52 (35.4)	7 (4.3)	***p* < 0.001**
Tachypnea *n* (%)	15 (4.8)	15 (10.2)	0 (0)	***p* < 0.001**
Dyspnea *n* (%)	56 (18)	56 (38.1)	0 (0)	***p* < 0.001**
Chest Pain *n* (%)	56 (18)	56 (38.1)	0 (0)	***p* < 0.001**
Co-morbidities *n* (%)				
No Comorbidities	269 (86.5)	106 (72.1)	163 (99.4)	***p* < 0.001**
Comorbidity = 1	27 (8.7)	26 (17.7)	1 (0.6)	***p* < 0.001**
Comorbidity ≥ 2	15 (4.8)	15 (10.2)	0 (0)	***p* < 0.001**
Site of Infection				
URTI *n* (%)	272 (87.5)	117 (79.6)	155 (94.5)	***p* < 0.001**
LRTI *n* (%)	27 (8.7)	20 (13.6)	7 (4.3)	***p* = 0.004**
Mixed URTI and LRTI *n* (%)	12 (3.9)	10 (6.8)	2 (1.2)	***p* = 0.015**

Abbreviations: IQR: Interquartile Range, URTI: Upper Respiratory Tract Infection, LRTI: Lower Respiratory Tract Infection.

**Table 2:** Comparison of Clinical Characteristics According to Detected Virus.

Variable	Adenovirus (*n* = 16)	Coronavirus (*n* = 19)	Human Metapneumovirus (*n* = 36)	Human Rhinovirus (*n* = 71)	Influenza A (*n* = 43)	Influenza B (*n* = 4)	Parainfluenza Virus (*n* = 30)	Enterovirus (*n* = 9)	RSV (*n* = 36)	SARS-CoV-2 (*n* = 47)	*p*-Value
Male Gender *n* (%)	8/16 (50)	4/19 (21.1)	13/36 (36.1)	26/71 (36.6)	18/43 (41.9)	2/4 (50)	13/30 (43.3)	2/9 (22.2)	14/36 (38.9)	20/47 (42.6)	*p* = 0.781
Age (mean, SD)	39.69 ± 13.07	51.47 ± 22.14	55.36 ± 20.18	50.96 ± 19.26	49.19 ± 16.54	37.5 ± 16.78	61.87 ± 22.17	46.47 ± 25.8	61.58 ± 18.3	59 ± 18.3	***p* < 0.001**
Fever *n* (%)	6/16 (37.5)	3/19 (15.8)	14/36 (38.9)	8/71 (11.3)	11/43 (25.6)	2/4 (50)	11/30 (36.7)	4/9 (44.4)	7/36 (19.4)	10/47 (21.3)	***p* = 0.021**
Cough *n* (%)	2/16 (12.5)	4/19 (21.1)	9/36 (25)	12/71 (16.9)	7/43 (16.3)	1/4 (25)	6/30 (20)	3/9 (33.3)	8/36 (22.2)	7/47 (14.9)	*p* = 0.926
Tachypnea *n* (%)	0/16 (0)	0/19 (0)	2/36 (5.6)	2/71 (2.8)	1/43 (2.3)	0/4 (0)	2/30 (6.7)	3/9 (33.3)	3/36 (8.3)	2/47 (4.3)	***p* = 0.016**
Dyspnea *n* (%)	2/16 (12.5)	2/19 (10.5)	11/36 (30.6)	8/71 (11.3)	6/43 (14)	1/4 (25)	7/30 (23.3)	3/9 (33.3)	8/36 (22.2)	8/47 (17)	*p* = 0.338
Chest Pain *n* (%)	2/16 (12.5)	2/19 (10.5)	11/36 (30.6)	8/71 (11.3)	6/43 (14)	1/4 (25)	7/30 (23.3)	3/9 (33.3)	8/36 (22.2)	8/47 (17)	*p* = 0.338
No Comorbidities *n* (%)	15/16 (93.8)	16/19 (84.2)	28/36 (77.8)	64/71 (90.1)	39/43 (90.7)	4/4 (100)	26/30 (86.7)	7/9 (77.8)	29/36 (80.6)	41/47 (87.2)	*p* = 0.656
Comorbidities = 1 *n* (%)	0/16 (0)	2/19 (10.5)	5/36 (13.9)	5/71 (7)	4/43 (9.7)	0/4 (0)	1/30 (3.3)	1/9 (11.1)	4/36 (11.1)	5/47 (10.6)	*p* = 0.824
Comorbidities ≥ 2 *n* (%)	1/16 (6.2)	1/19 (5.3)	3/36 (8.3)	2/71 (2.8)	0/43 (0)	0/4 (0)	3/30 (10)	1/19 (11.1)	3/36 (8.3)	1/47 (2.1)	*p* = 0.506
Upper Respiratory Tract Infection *n* (%)	13/16 (81.2)	16/19 (84.2)	32/36 (88.9)	61/71 (85.9)	37/43 (86)	4/4 (100)	26/30 (86.7)	7/9 (77.8)	30/36 (83.3)	46/47 (97.9)	*p* = 0.574
Lower Respiratory Tract Infection *n* (%)	2/16 (12.5)	3/19 (15.8)	2/36 (5.6)	8/71 (11.3)	4/43 (9.3)	0/4 (0)	2/30 (6.7)	1/9 (11.1)	4/36 (11.1)	1/47 (2.1)	*p* = 0.742
Mixed Upper and Lower Respiratory Tract Infection *n* (%)	1/16 (6.2)	0/19 (0)	2/36 (5.6)	2/71 (2.8)	2/43 (4.7)	0/4 (0)	2/30 (6.7)	1/9 (11.1)	2/36 (5.6)	0/47 (0)	*p* = 0.759

Abbreviations: *n*: Number, SD: Standard Deviation, URTI: Upper Respiratory Tract Infection, LRTI: Lower Respiratory Tract Infection, RSV: Respiratory Syncytial Virus, SARS-CoV-2: Severe Acute Respiratory Syndrome Coronavirus-2.

**Table 3:** Univariate and Multivariate logistic regression analysis of predictors for hospital admission.

Variable	Univariate Analysis			Multivariate Analysis		
	OR	95% CI	*p* Value	Adjusted OR	95% CI	*p* Value
Demographic characteristics						
Gender (Male)	1.660	1.048–2.630	*p* = 0.031	1.101	0.577–2.103	*p* = 0.770
Age (over 60)	5.961	3.542–10.033	*p* < 0.001	6.110	3.046–12.256	***p* < 0.001**
Clinical Manifestations						
Fever	19.841	8.708–45.207	*p* < 0.001	20.502	7.067–59.475	***p* < 0.001**
Cough	12.277	5.358–28.132	*p* < 0.001	3.999	1.280–12.494	***p* = 0.017**
Site of Infection (Reference Upper Respiratory Tract Infection)						
Lower Respiratory Tract Infection	3.785	1.549–9.250	*p* = 0.004	11.109	3.609–34.196	***p* < 0.001**
Mixed Upper and Lower Respiratory Tract Infection	6.624	1.424–30.807	*p* = 0.016	22.285	4.318–115.002	***p* < 0.001**
Confirmed Viral Pathogen						
Adenovirus	1.463	0.531–4.032	*p* = 0.462	0.555	0.113–2.721	*p* = 0.468
Coronavirus	0.377	0.132–1.074	*p* = 0.068	0.066	0.011–0.387	***p* = 0.003**
Human Metapneumovirus	1.457	0.724–2.930	*p* = 0.291	0.470	0.137–1.614	*p* = 0.230
Human Rhinovirus	0.448	0.257–0.783	***p* = 0.005**	0.235	0.086–0.644	***p* = 0.005**
Influenza A	0.776	0.404–1.488	*p* = 0.445	0.261	0.084–0.808	***p* = 0.02**
Influenza B	1.117	0.155–8.033	*p* = 0.912	0.410	0.021–8.158	*p* = 0.559
Parainfluenza Virus	0.974	0.458–2.071	*p* = 0.945	0.151	0.041–0.560	***p* = 0.005**
Enterovirus	2.284	0.561–9.300	*p* = 0.249	1.249	0.176–8.880	*p* = 0.824
RSV	1.886	0.926–3.839	*p* = 0.08	0.632	0.202–1.983	*p* = 0.432
SARS-CoV-2	1.796	0.955–3.375	*p* = 0.069	**-**		

Abbreviations: OR: Odds Ratio, CI; Confidence Interval, URTI: Upper Respiratory Tract Infection, LRTI: Lower Respiratory Tract Infection, RSV: Respiratory Syncytial Virus, SARS-CoV-2: Severe Acute Respiratory Syndrome Coronavirus-2.

## Data Availability

Data can be made available upon request.

## Supplementary Materials

The following supporting information can be downloaded at: https://www.mdpi.com/article/10.3390/v18010135/s1, Figure S1: Study Flowchart.

## Abbreviations

The following abbreviations are used in this manuscript:

ARDS	Acute Respiratory Distress Syndrome
ATS	American Thoracic Society
CDC	Centers for Disease Control and Prevention
CI	Confidence Interval
COPD	Chronic Obstructive Pulmonary Disease
COVID-19	Coronavirus Disease 2019
ECDC	European Centre for Disease Prevention and Control
ED	Emergency Department
EMR	Electronic Medical Record
hMPV	Human Metapneumovirus
HRV	Human Rhinovirus
ICU	Intensive Care Unit
IDSA	Infectious Diseases Society of America
IQR	Interquartile Range
LRTI	Lower Respiratory Tract Infection
OR	Odds Ratio
PCR	Polymerase Chain Reaction
PORT	Patient Outcomes Research Team
PSI	Pneumonia Severity Index
RSV	Respiratory Syncytial Virus
RTI	Respiratory Tract Infection
SARS-CoV-2	Severe Acute Respiratory Syndrome Coronavirus 2
SD	Standard Deviation
URTI	Upper Respiratory Tract Infection
