# Supplementary figures and images for: Clinical, Demographic, and Virological Predictors of Hospital Admission in Patients with Acute Viral Respiratory Infections: A Retrospective Observational Study
Source: Viruses. 2026 Jan 21;18(1):135. doi: 10.3390/v18010135 (PMC12846340; doi:10.3390/v18010135)

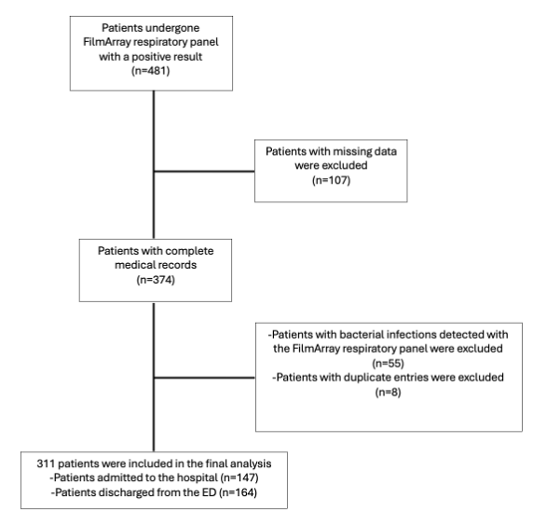

Supplement: Supplementary file 1 [file viruses-18-00135-s001.zip › viruses-4084189-supplementary.png]
